# An Augmented Reality Serious Game for Children’s Optical Science Education: Randomized Controlled Trial

**DOI:** 10.2196/47807

**Published:** 2024-02-01

**Authors:** Bo Liu, Xinyue Wan, Xiaofang Li, Dian Zhu, Zhao Liu

**Affiliations:** 1 School of Design, Shanghai Jiao Tong University, Shanghai, China

**Keywords:** augmented reality, serious game, science education, childhood education, cognition, children, scientific cognition, cognitive process, effectiveness

## Abstract

**Background:**

Knowledge construction in the context of children’s science education is an important part of fostering the development of early scientific literacy. Nevertheless, children sometimes struggle to comprehend scientific knowledge due to the presence of abstract notions.

**Objective:**

This study aimed to evaluate the efficacy of augmented reality (AR) games as a teaching tool for enhancing children’s understanding of optical science education.

**Methods:**

A total of 36 healthy Chinese children aged 6-8 years were included in this study. The children were randomly divided into an intervention group (n=18, 50%) and a control group (n=18, 50%). The intervention group received 20 minutes of AR science education using 3 game-based learning modules, whereas the control group was asked to learn the same knowledge for 20 minutes with a non-AR science learning app. Predict observe explain tests for 3 topics (animal vision, light transmission, and color-light mixing) were conducted for all participants before and after the experiment. Additionally, the Intrinsic Motivation Inventory, which measures levels of interest-enjoyment, perceived competence, effort-importance, and tension-pressure, was conducted for children after the experiment.

**Results:**

There was a statistically significant difference in light transmission (*z*=−2.696; *P*=.008), color-light mixing (*z*=−2.508; *P*=.01), and total predict observe explain test scores (*z*=2.458; *P*=.01) between the 2 groups. There were also variations between the groups in terms of levels of interest-enjoyment (*z*=−2.440; *P*=.02) and perceived competence (*z*=−2.170; *P*=.03) as measured by the Intrinsic Motivation Inventory.

**Conclusions:**

The randomized controlled trial confirmed that the AR-based science education game we designed can correct children’s misconceptions about science and enhance the effectiveness of science education.

## Introduction

Children’s level of scientific concept generation is representative of their inquiry, comprehension, and application of natural events and phenomena and reveals their cognitive capacities and developmental stages [1]. Knowledge construction in children’s science education contributes to early scientific literacy development, which improves children’s cognitive level by enhancing thinking skills, and is being emphasized by scholars and parents [2]. Traditionally, children build domain knowledge in science through films, literature, and lectures in science education [3]. Although some forms of educational learning are accessible, they often uses a monotonous instructional format, and confusing content hinders the transmission of scientific knowledge [4].

Serious games provide a more engaging interactive environment and an accessible cognitive framework to facilitate effective learning [5]. Studies have shown that serious games have more effective learning outcomes than traditional methods of science education (eg, face-to-face lectures and book-based knowledge transfer) [6,7]. It is suitable for children’s investigation of natural phenomena because the game’s visual design simulates paranormal phenomena that cannot be produced in real life. Lester et al [8] constructed virtual environments that generate natural phenomena, allowing children to assume roles in open-world environments and to freely rely on their knowledge of the geography and biology of natural environments. Laine et al [9] permitted children to interact with hosts in virtual narrative game scenarios and to investigate the geometry of the virtual environment with the protagonist. The concept of light, a prevalent natural phenomenon, was selected as the subject of this research to explore its design for enhancing children’s cognitive abilities. Optical science education programs are still presented in a 2D format, which has been demonstrated to be ineffective [10].

Due to the spatial complexity and abstract nature of optics, it is challenging to accurately convey knowledge through flat visual representations [11]. Therefore, it is necessary to blur the boundaries between the 3D real world and the 2D digital world to reduce the distance between children’s learning of science concepts and their learning environments [12]. 3D representations and interactions in augmented reality (AR) games have the potential to enhance spatial cognition, thereby facilitating children’s comprehension of spatially abstract scientific concepts [13], such as simulating the movement of the sun in a classroom environment [14]. Sahin and Yilmaz [15] demonstrated that students who used AR technology to improve their science literacy performed better on tests than those who learned using traditional methods. This is as a result of AR technology’s ability to enhance the dynamic potential of human consciousness to comprehend the science learning process [16]. In addition, motivational improvement was mentioned as one of the frequently observed AR outcomes [17]. Using AR apps increased student motivation relative to other instructional aids [18]. Our study investigated whether designing optical science education with more comprehensible 3D interactions for children can enhance science education and promote children’s motivation.

The study designed the “AR Serious Game for Optical Science” and conducted a randomized controlled trial to determine the efficacy of this AR game product in enhancing children’s science education. The primary objective of this study was to validate the efficacy of AR science education games for children; the secondary objective was to investigate the intrinsic motivation of children toward them.

## Methods

### Study Design

Guardians of children with independent mobility provided informed written consent for their participation in the study. Participants were randomly assigned to the intervention and control groups using a randomization list, which was maintained by members of the study group uninvolved in any other aspect of the study. Participants’ guardians received and opened opaque, sealed envelopes containing group assignments following the initial evaluation. The evaluator in charge of assessing the results of the AR science education course had no access to participant information or group assignment.

Sample size calculations were performed using PASS software (NCSS LLC) based on the predict observe explain (POE) test scores from the preintervention questions. Group sample sizes of 18 and 18 achieve 90.118% power to reject the null hypothesis of equal means, when the population mean difference is μ1 − μ2 = 3.2 − 1.0 = 2.2, with SDs of 2.0 and 1.9 for the 2 groups and with a significance level (α) of .05 using a bilateral, 2-sample, equal-variance, 2-tailed *t* test.

### Participants

A total of 36 Chinese children (aged 6-8 y) were recruited from Jiangyin Children’s Education Center and Jiangyin Wuxi Community in Jiangsu Province and divided into the intervention (n=18, 50%) and control (n=18, 50%) groups.

### AR Science Education Game Design

During the learning phase, children are required to engage in physical activities, such as walking around with a handheld device, to interact with the AR scene’s content to discover what is unique about the light phenomenon. When children touch the interactive points, the content is explained by animation and voice-overs. This study developed several interactive approaches for children within AR games, such as through in-game visual representations, speech, and interactive methods, which permit children to connect game content to unfamiliar information as they explore. The advantages are as follows: (1) children can use more familiar physical activities with light concepts to establish metaphorical mappings related to orientation, not just gestural touch; (2) rendering light with 3D attributes in the real world reduces the cognitive load generated by children’s linkage of abstract knowledge and the real phenomena; and (3) adding various kinds of digital augmentation effects in the AR scene helps children understand the concepts. The project created 3 games based on the characteristics of scientific understanding (Multimedia Appendix 1 and Figure S1 in Multimedia Appendix 2 [9,14,19-28]).

**Figure 1.**
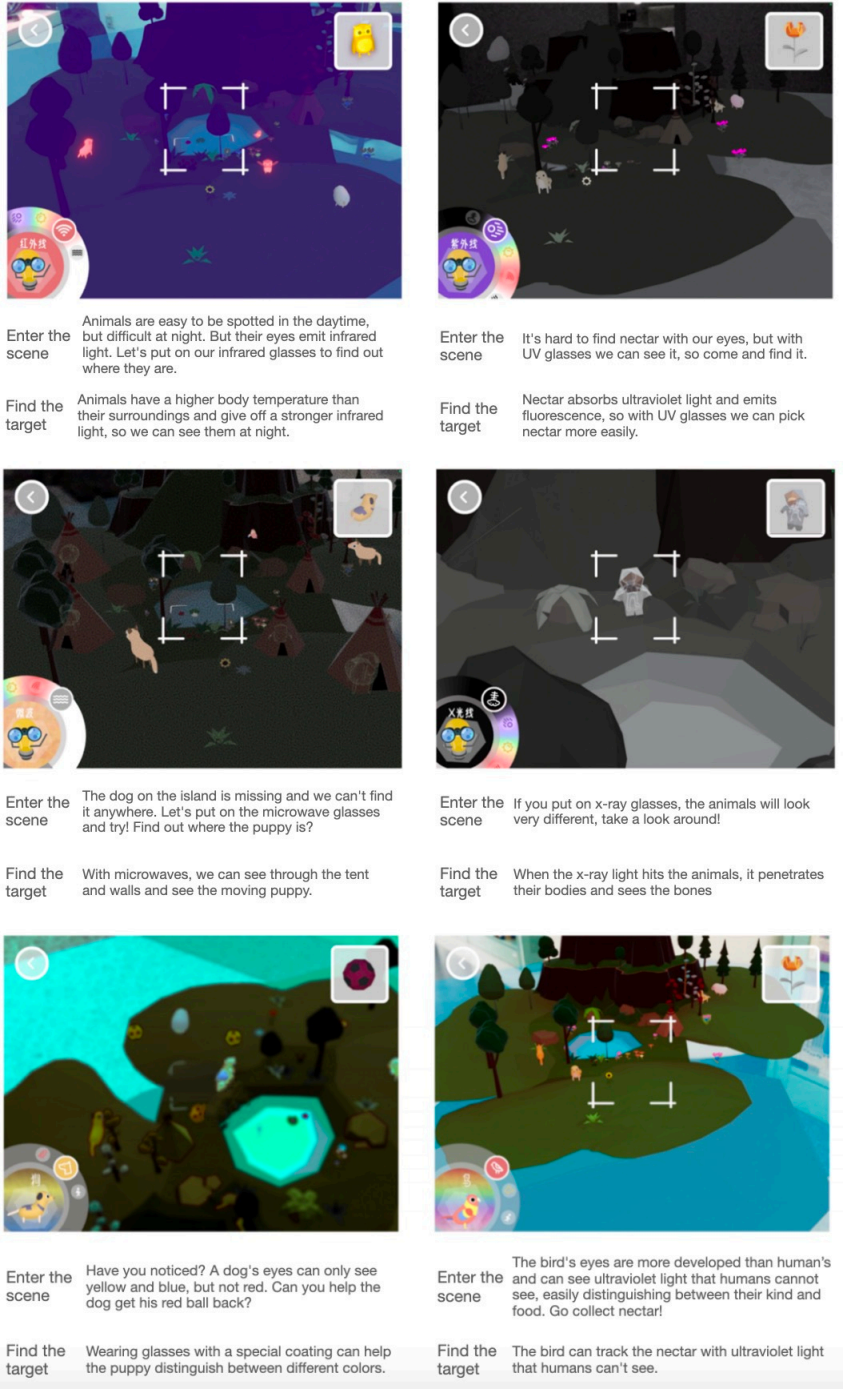
Animal vision augmented reality (AR) game introduction.

Game 1 introduces children to the fundamentals of animal vision (Figure 1). Animal vision concepts are investigated through AR scenes. By clicking on the icons in the lower-left corner, the game transforms to an animal simulation. In each scenario, a voice-over narration instructs children to identify the visual differences between the animal and the human. When the handheld device is trained on a specific target, a voice-over narration and feedback animation will play.

In the design of the interaction mode, 3 display modes were established for the game’s interactive elements: far, medium, and near (hybrid camera mode). The concept of invisible light is introduced to children in greater detail based on the ray distance between the device’s camera and the target element. The far view provides children with an intuitive impression of the invisible light’s overall effect; the medium view uses transition animation to illustrate the invisible light’s characteristics; and the near view uses special effect particles to illustrate the invisible light’s trajectory.

**Figure 2.**
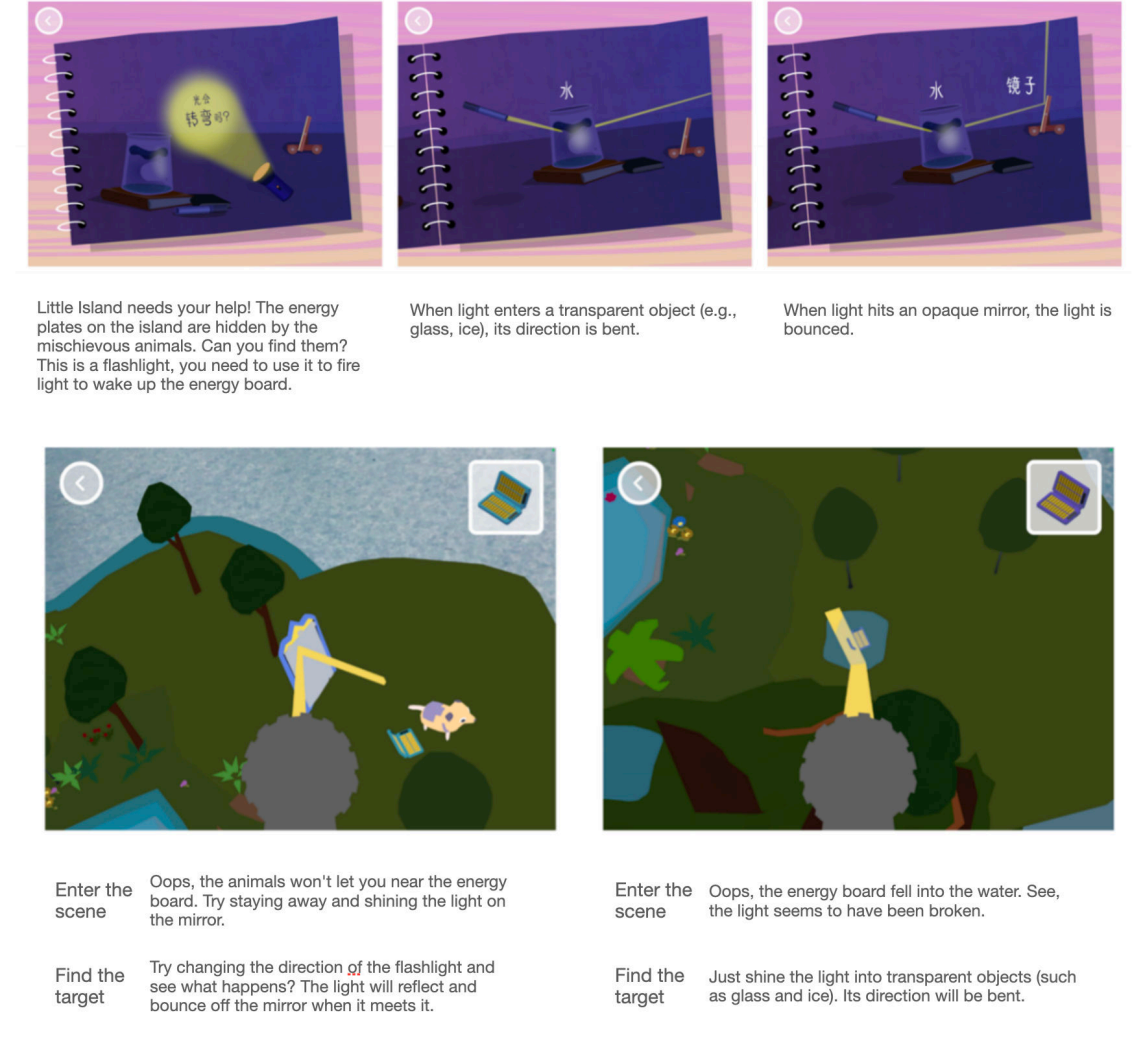
Light transmission augmented reality (AR) game introduction.

Game 2 introduces children to light transmission–related concepts (Figure 2). In the AR scenario, children navigate the environment with a handheld device and activate energy panels by interacting with flat mirrors and optics. By targeting AR-enhanced prop objects and manipulating the angle of light emission to investigate how light propagates, voice-over explanations and feedback animations are activated.

According to the voice-over prompts, children can hold the device and manipulate the flashlight from a first-person perspective (spatial exploration mode) as part of the interactive design. They then complete 3 steps: locating the interactive elements (mirrors, ice crystals, etc), adjusting the flashlight’s tilt angle, and using the flashlight to complete the light-up task. The progression encourages children to investigate the principles of light transmission through the game.

**Figure 3.**
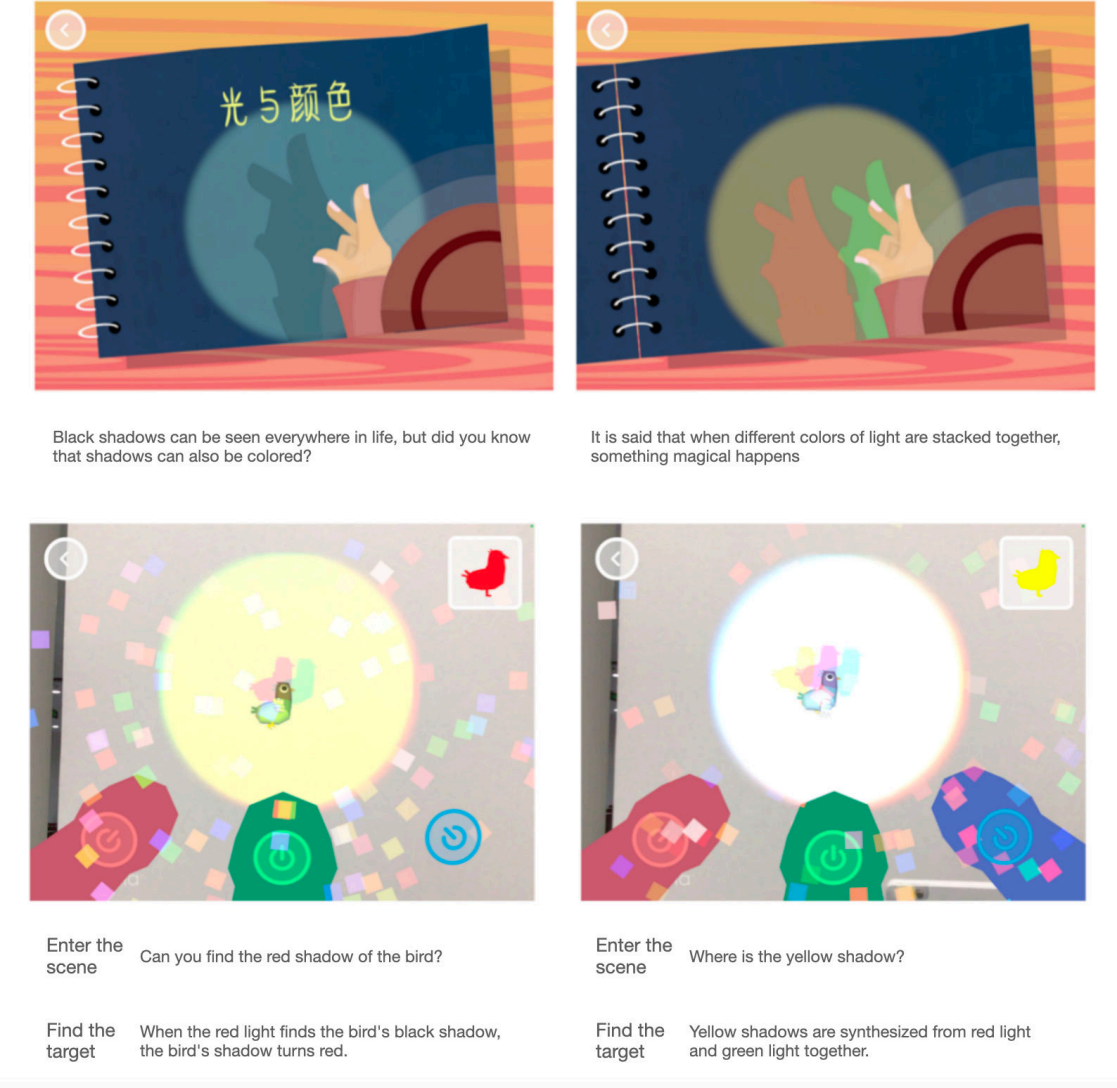
Color-light mixing augmented reality (AR) game introduction.

Game 3 introduces children to color-light mixing concepts (Figure 3). Children were instructed to walk around with the device in hand and explore the color changes of props such as AR-enhanced birds, which are illuminated with various colors of lights. Collecting the target color’s shadow initiates a voice-over explanation and feedback animation.

Regarding the interactive design, children need to hold the device to illuminate the creatures and cast shadows on the present wall, and then they need to press the button to turn the light on and off (projection irradiation mode). The objective of the game required children to perform single-color illumination, 2-color mixing, and 3-color mixing to achieve the desired hue. In another vibrant nursery game, children were instructed to move plants to receive various colors of light and to observe the plants’ root elongation and leaf dispersal.

This game design used Unity 3D (Unity Technologies) as the development engine, and the app was installed on an Apple iPad (2018) with a screen resolution of 2048×1536 (264 pixels per inch). The AR component made use of the Vuforia AR SDK (Parametric Technology Corporation) to accomplish the fundamental duties of plane identification and virtual object generation. The interaction section used lens focus to determine the interactions; when the device camera’s output rays collide with the target virtual object and the distance is close, it is deemed to have located the target effectively. To imitate the illusion of invisible light, Unity’s *Post Processing* module was applied to the camera filter. The principle entailed presenting the camera screen into the buffer of Unity and applying filters and effects prior to displaying it; it can be applied to both the camera screen and the virtual item.

### Procedure

This experiment was a randomized controlled trial, and the participants were randomly separated into the intervention group and the control group. The random numbers were generated by applying the SAS software analysis system (SAS Institute) on a computer simulation, and no experimental group was allowed to be selected at random. Every child was tested in the company of a guardian and 2 researchers.

The independent variable was the type of game (an optical science education app called “Light and Color” or the AR game we designed; see Figure S2 in Multimedia Appendix 2 for a comparison of the differences between the 2 games). The dependent variables for both intervention and control group participants were the differences between the pre- and posttest results of the POE tests and the children’s motivation to play the game. To create control variables for the experiment, both games included the topics of animal vision, light transmission, and color-light mixing, and neither game involved a human teacher. In addition, there were no significant sex (*P*=.49) or age (*P*=.67) differences between the 2 groups.

### Intervention Group

Before the test started, the researcher provided the basic information of the experiment to the participants, including the test topic, test technique, test time, and other information. The participants were asked to complete a cognitive exam on the notion of light and perform a POE test for each topic to find out how well they comprehend the content, without being told whether their answers were correct.

After completing the pretest, intervention group participants were instructed to complete the 3 game-based learning modules of the AR science education app on the iPad regarding animal vision, light transmission, and color-light mixing. On their initial encounter with the game, respondents were given around 10 minutes to comprehend its mechanics. The intervention group’s total learning time was limited to 20 minutes, the testing process was completed under the supervision of the instructor and the experimenter, and the children’s behavioral characteristics were recorded. During the experiment, the participants were not disturbed in any way; researchers only intervened when they faced difficulties or requested assistance. The participants were given a 15-minute respite at the conclusion of the trial to take another POE test. Before and after the experiment, each participant’s performance on the game was recorded. The researcher then read aloud and described the items on the intrinsic motivation and cognitive load scales to the participants, who scored the scale items using a 5-point “smiley face” scale.

### Control Group

**Figure 4.**
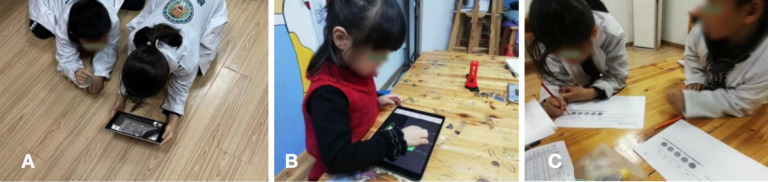
Photos of the experimental process: (A) the process of using the augmented reality (AR) game for participants in the intervention group; (B) the process of using the “Light and Color” app game for participants in the control group, and (C) the process of filling out the questionnaire by the participants.

The control group was also introduced to the experiment and given a preintervention POE test to assess their prior knowledge of the learning material. The control group completed the same 3 game courses for a maximum of 20 minutes using the non-AR app “Light and Color” after completing the pretest. The participants took a 15-minute break at the conclusion of the trial to complete another POE test and the Intrinsic Motivation Inventory (IMI) scale (Figure 4).

### Evaluation Metrics

The study was validated based on several experiments.

The POE test is commonly used in science classes and tries to expose students’ expectations about certain events and the rationale for these predictions [29]. It is used to demonstrate scientific experiments to pupils and is advantageous for fostering children’s critical thinking and assessing students’ grasp of scientific topics. The investigator then displays the relevant physical events to the students using basic prop materials after requiring the students to independently determine the correct answers to the questionnaire along with their justifications. Finally, students are instructed to alter or supplement their explanations in light of the observations. Since children may appear to be able to answer the question properly but not comprehend the reasoning behind it, for each topic, it is possible that they do not comprehend the underlying concept. In this study, individuals’ accurate answers and explanations were recorded, and different situations were rated differently based on a 2-tier test [30] (Table 1). This scoring method is frequently used to evaluate students’ conceptual understanding [31]. The outcomes were categorized as correct answer+correct explanation, correct answer+incorrect explanation, incorrect answer+correct explanation, and incorrect answer+incorrect explanation. Each topic’s overall score was included in the subsequent analysis. To avoid disruptions caused by children’s memorization of answers, the experimental posttest questionnaire in this study was different from the pretest questionnaire but was founded on the same scientific concepts. The examination topics are provided in Table S1 in Multimedia Appendix 2.

**Table 1.** Two-tier test assessment criteria.

Level of conceptual understanding	Score
Correct answer+correct explanation	2
Correct answer+incorrect explanation	1
Incorrect answer+correct explanation	1
Incorrect answer+incorrect explanation	0

Due to the young age of the study participants, the simplified version of the IMI adapted by Vos et al [32] was selected for this research. It was developed under game conditions with 3 subscales: interest-enjoyment, perceived competence, and effort-importance, to assess the perceived levels of motivation, enjoyment, and perceived difficulty of the participants. To investigate the negative emotions of children using the AR game, the study inserted questions from the original scale’s tension-stress section [33] (Table S2 in Multimedia Appendix 2). Participants were asked to rate the extent to which they concurred with the statement using a 5-point Likert scale depicting 5 smiling faces. A score of 5 indicated that the child participant strongly agreed with the statement. To minimize the effect of differences in reading ability, the researcher read the questionnaire audibly to the child participants, who then completed the questionnaire independently.

### Ethical Considerations

The study was approved by the Human Research Ethics Committee of Shanghai Jiao Tong University (H2022041I) in China. Informed consent was signed by guardians and the data were deidentified. A toy with a value of CNY ¥50 (US $7.01) was provided as compensation.

## Results

**Figure 5.**
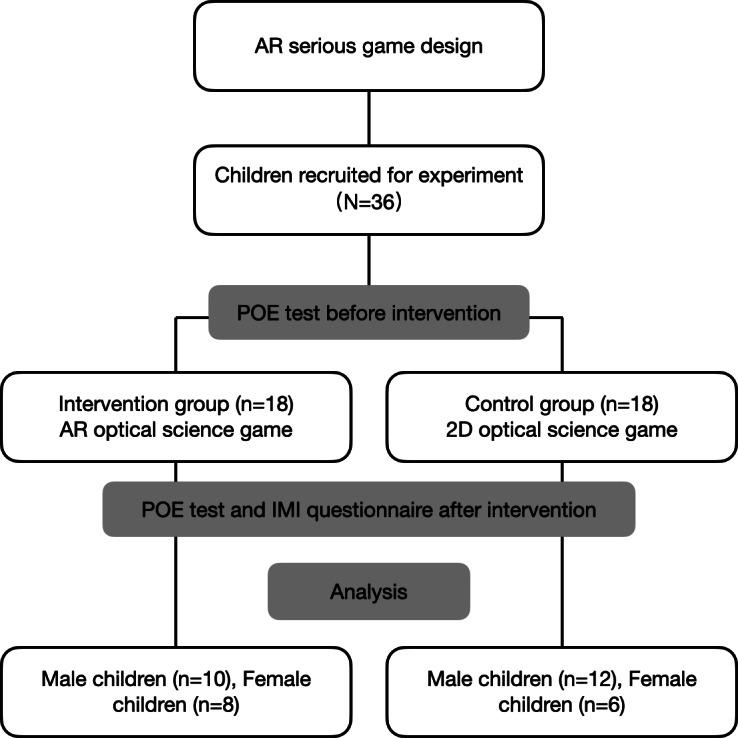
CONSORT (Consolidated Standards of Reporting Trials) flow diagram. AR: augmented reality; IMI: Intrinsic Motivation Inventory; POE: predict observe explain.

A total of 36 healthy Chinese children aged 6-8 years were recruited in May 2022, including 22 male and 14 female children, all of whom participated in the experiment with the consent of their guardians and of their own volition. The 36 participants were randomly assigned to the intervention group (n=18, 50%) and the control group (n=18, 50%), with the mean age of the intervention group being 7.16 (SD 0.76) years and that of the control group being 7.06 (SD 0.78) years. Baseline demographic data and POE test scores for the intervention and control groups are shown in Table 2. The statistical analysis revealed that there was no statistically significant distinction observed between the 2 groups across all variables (all *P*>.05). This suggests that the intervention and control groups exhibited a similar overall comprehension level prior to the commencement of the trial. The experimental procedure is provided in Figure 5.

**Table 2.** Baseline data for the intervention and control groups.

Variable	Intervention group^a^ (n=18)	Control group^b^ (n=18)	*z* score or chi-square (*df*)	*P* value^c^
Male sex, n (%)	10 (56)	12 (67)	0.467 (1)^d^	.49
Age (y), mean (SD)	7.17 (0.76)	7.06 (0.78)	−0.421^e^	.67
POE^f^ test score for animal vision, mean (SD)	1.83 (1.10)	1.94 (1.21)	−0.296^e^	.77
POE test score for light transmission, mean (SD)	2.83 (1.72)	2.67 (2.20)	−0.437^e^	.66
POE test score for color-light mixing, mean (SD)	1.50 (1.09)	1.94 (1.16)	−1.031^e^	.30
Total POE test score, mean (SD)	6.17 (2.28)	6.56 (2.12)	−0.273^e^	.78

a Augmented reality game.

b Non–augmented reality game.

c Mann-Whitney *U* test and *χ*2.

d Chi-square value.

e *z* score.

f POE: predict observe explain.

The results of the normality test revealed a nonnormal distribution of the data (Table S3 in Multimedia Appendix 2). Consequently, the researchers conducted a paired-sample Wilcoxon rank sum test to compare the pre- and posttest findings of the intervention and control groups to assess any differences between the 2 groups. The results shown in Table 3 demonstrate notable fluctuations in both light transmission (*z*=−2.696; *P*=.008) and total POE test scores (*z*=−2.458; *P*=.01). Nevertheless, the results of the study indicate that there was no statistically significant advantage observed in animal vision (*z*=−0.847; *P*=.42) and color-light mixing POE test scores (*z*=−0.782; *P*=.46) as a result of the AR game intervention. It should be noted, however, that there was an improvement in scores following the intervention.

**Table 3.** Between-group differences between the intervention and control groups on each of predict-observe-explain (POE) test (pre- and posttests).

POE test score		Intervention group^a^ (n=18), mean (SD)	Control group^b^ (n=18), mean (SD)	Difference, mean (95% CI)	*z* score	*P* value^c^
**Animal vision**				0.36 (−0.71 to 1.43)	−.847	.42
	Pretest	1.83 (1.10)	1.94 (1.21)			
	Posttest	2.33 (1.14)	2.17 (0.99)			
**Light transmission**				0.97 (−0.37 to 2.31)	−2.696	.008
	Pretest	2.83 (1.72)	2.67 (2.2)			
	Posttest	4.44 (1.76)	3.00 (1.88)			
**Color-light mixing**				0.72 (−0.44 to 1.88)	−0.782	.46
	Pretest	1.50 (1.09)	1.94 (1.16)			
	Posttest	2.39 (1.24)	2.50 (1.04)			
**Total**				2.06 (−0.1 to 4.22)	−2.458	.01
	Pretest	6.17 (2.28)	6.56 (2.12)			
	Posttest	9.17 (2.48)	7.67 (1.71)			

a Augmented reality game.

b Non–augmented reality game.

c Mann-Whitney *U* test.

In this study, subjective IMI scale values acquired during the trial were statistically analyzed. It was observed that the different groups showed significant variability in levels of interest-enjoyment (*z*=−2.440; *P*=.02) and perceived competence (*z*=−2.170; *P*=.03; Table 4), whereas significant differences were not observed in levels of effort-importance (*z*=−1.310; *P*=.20) and tension-pressure (*z*=−0.733; *P*=.48).

**Table 4.** Comparison of Intrinsic Motivation Inventory (IMI) variability between the intervention and control groups.

IMI subscale	Intervention group^a^ (n=18), mean (SD)	Control group^b^ (n=18), mean (SD)	*z* score	*P* value^c^
Interest-enjoyment	20.28 (2.72)	18.00 (2.57)	−2.440	.02
Perceived competence	18.83 (3.05)	16.33 (3.34)	−2.170	.03
Effort-importance	12.89 (1.97)	11.83 (2.57)	−1.310	.20
Tension-pressure	10.44 (2.33)	9.77 (2.90)	−0.733	.48

a Augmented reality game.

b Non–augmented reality game.

c Mann-Whitney *U* test.

## Discussion

### Principal Findings

The integration of science education into the foundational education of children aims to systematically cultivate their abilities in inductive and deductive thinking [34]. Serious games have demonstrated efficacy in enhancing teaching and learning outcomes within the contemporary domain of children’s science education [7]. AR technology has garnered growing interest in the realm of serious game design in recent times due to its ability to visually represent scientific processes that are not easily observable in real-life situations [35]. Further, the incorporation of AR technology into mobile devices has resulted in widespread adoption, facilitating the implementation of many apps [17]. Nevertheless, there is a lack of comprehensive study and experimentation to substantiate the efficacy of AR design in the realm of children’s science education. Consequently, a series of AR science instructional games were developed, focusing on the comprehension of light principles. The objective was to assess the efficacy of the games and the degree of intrinsic motivation of the students. The results showed that children who participated in the AR science game had substantially higher POE test scores and conceptual understanding of light propagation than the control group.

The study revealed that children exhibited varying levels of comprehension in relation to light concepts across diverse themes. Reliable between-group differences were detected among the topics of light propagation. The rationale behind the use of AR lies in its inherent benefits, which include the ability for children to engage in physical activity while delving into a more comprehensive exploration as compared to 2D games. Additionally, AR technology facilitates the rendering of real-world light phenomena, as supported by previous studies [36,37]. Our game was developed with the purpose of creating a metaphorical representation of concepts connected to light orientation using gesture-touch interactions. It aims to alleviate the cognitive burden experienced by children when trying to connect abstract information about light with real-world light occurrences. This is achieved by incorporating 3D properties of lighting effects into the game. Furthermore, a notable increase in the game scores of the intervention group was noticed across all topics. This observation serves as evidence for the beneficial influence of AR games on children’s conceptual transformation during the process of acquiring scientific knowledge. Our game also serves as a means of scientific investigation, necessitating active engagement from the children. Certain children had not before contemplated the underlying mechanisms responsible for commonplace visual occurrences. The stimulation of their drive to study and their high curiosity played a significant role in facilitating their conceptual shift and fostering the development of scientific thinking [38]. Nevertheless, the intervention group did not exhibit any noteworthy disparity compared to the control group in relation to the topics of animal vision and color-light mixing. The limited influence of the different interactive designs, specifically mixed camera mode and projected lighting mode, on children’s cognition may account for this disparity when compared to the visual representation format in the 2D game.

Intrinsic motivation is a potent factor that influences performance, learning persistence, and productivity [39]. Children in the intervention group demonstrated greater interest and enjoyment in intrinsic motivation than those in the control group, and they demonstrated an ability to embrace and comprehend the causes of certain light phenomena.

AR imparts scientific information that challenges children’s prior knowledge and stimulates their interest. Consequently, it can arouse interest in the principles and stimulate active thought [35]. During the test, we observed that participants had a keen interest in the game and avidly explored the interface’s interactive elements. Moreover, there was a significant difference in perceptual ability between the intervention and control groups. We believe that this difference stems from the fact that AR games, created by adding 3D virtual objects to real-world images, can better facilitate children’s understanding of complex concepts [15]. However, we also found that the intervention group showed some stress toward AR games. Children have a period of adjustment for things to which they are not accustomed to, as evidenced by their inattention and attempts to communicate with the observer when they encounter difficulties in the game [17]. Future research can therefore concentrate on how to provide prompts even when encountering difficulties.

The researchers observed that the children’s engagement in gameplay facilitated their conscious observation of light occurrences in their daily lives, resulting in a modest improvement in their comprehension during the final phase of the tests. Furthermore, when the optical principles pertaining to linear propagation, reflection, and refraction became increasingly complex, it became more challenging for the children to comprehend, leading to confusion in certain preintervention participants regarding the distinctions between these concepts. It is important to acknowledge that when a child misinterprets the dynamic effects, animation, or creative expression of a game feature, the game can potentially facilitate the development of novel alternative understanding. Fortunately, the occurrence of this scenario was limited in the 2 assessment tests conducted during the formal experiment.

The strengths and weaknesses of our study in comparison with other studies is shown in Table S4 in Multimedia Appendix 2.

In summary, the integration of AR into educational games has the potential to enhance children’s science education by offering a more immersive and engaging learning experience. This approach also may address the challenges associated with inadequate education and the lack of motivation among children to explore scientific subjects.

### Conclusions and Limitations

The results suggest that the use of AR serious games can effectively motivate children to undergo conceptual shifts during the initial phases of science education. This, in turn, leads to an improved level of comprehension of scientific material. Furthermore, it is expected that these positive outcomes can be replicated in future preschool science education settings. This randomized controlled trial provides confirmation that the science education game we developed, using AR technology, has the potential to rectify children’s misconceptions regarding scientific concepts and improve the overall efficacy of science teaching.

However, there are also some limitations. First, the sample size used in the study was limited, and the sample population was mainly from the more resource-rich region of Jiangsu Province, China. Consequently, it is challenging to ascertain the presence of regional variations in other geographical areas. Prospective studies with large samples are needed to further confirm the results, and the results can be improved by considering gender, family upbringing, and children’s interest preferences in subsequent studies. Second, AR apps require a lot of attention and can be a distraction. It can cause students to ignore instructions or important stages of the experience. In addition, as the situation appeared in the pre-experiment, the game as a teaching tool may generate new misconceptions if the child misinterprets the content of the game. Finally, the existing game conveys scientific concepts mostly through voice-over prompts, which are insufficient to grab the children’s attention, and children may be distracted and lose essential information during the voice-over prompts.

## Supplementary material

10.2196/47807 Multimedia Appendix 1 The designed augmented reality game.

10.2196/47807 Multimedia Appendix 2 Supplementary tables and figures.

10.2196/47807 Checklist 1 CONSORT eHEALTH Checklist.

## Abbreviations

AR: augmented reality
IMI: Intrinsic Motivation Inventory
POE: predict observe explain

## References

[1] Duit R, Treagust DF (2003). Conceptual change: a powerful framework for improving science teaching and learning. Int J Sci Educ.

[2] Adey P, UNESCO International Bureau of Education (1999). The science of thinking, and science for thinking: a description of cognitive acceleration through science education (CASE). UNESCO.

[3] Gopnik A (2012). Scientific thinking in young children: theoretical advances empirical research, and policy implications. Science.

[4] Magnusson S, Krajcik J, Borko H, Gess-Newsome J, Lederman NG (1999). Examining Pedagogical Content Knowledge.

[5] Nazry NNM, Romano DM (2017). Mood and learning in navigation-based serious games. Comput Hum Behav.

[6] Malaquias RF, Malaquias FFO, Hwang Y (2018). Understanding technology acceptance features in learning through a serious game. Comput Hum Behav.

[7] Zhonggen Y (2019). A meta-analysis of use of serious games in education over a decade. International Journal of Computer Games Technology.

[8] Lester JC, Spires HA, Nietfeld JL, Minogue J, Mott BW, Lobene EV (2014). Designing game-based learning environments for elementary science education: a narrative-centered learning perspective. Inf Sci.

[9] Laine TH, Nygren E, Dirin A, Suk HJ (2016). Science Spots AR: a platform for science learning games with augmented reality. Education Technology Research and Development.

[10] Wang M, Zheng X (2021). Using game-based learning to support learning science: a study with middle school students. Asia-Pacific Education Researcher.

[11] Amin TG (2015). Conceptual metaphor and the study of conceptual change: research synthesis and future directions. Int J Sci Educ.

[12] Oranç C, Küntay AC (2019). Learning from the real and the virtual worlds: educational use of augmented reality in early childhood. International Journal of Child-Computer Interaction.

[13] Arici F, Yildirim P, Caliklar Ş, Yilmaz RM (2019). Research trends in the use of augmented reality in science education: content and bibliometric mapping analysis. Computers & Education.

[14] Tarng W, Ou KL, Lu YC, Shih YS, Liou HH (2018). A sun path observation system based on augment reality and mobile learning. Mobile Information Systems.

[15] Sahin D, Yilmaz RM (2020). The effect of augmented reality technology on middle school students’ achievements and attitudes towards science education. Computers & Education.

[16] Fleer M (2013). Affective imagination in science education: determining the emotional nature of scientific and technological learning of young children. Res Sci Educ.

[17] Garzón J, Pavón J, Baldiris S (2019). Systematic review and meta-analysis of augmented reality in educational settings. Virtual Reality.

[18] di Serio Á, Ibáñez MB, Kloos CD (2013). Impact of an augmented reality system on students’ motivation for a visual art course. Computers & Education.

[19] Nkadimeng M, Ankiewicz P (2022). The affordances of Minecraft Education as a game-based learning tool for atomic structure in junior high school science education. J Sci Educ Technol.

[20] Swacha J, Maskeliūnas R, Damaševičius R (2021). Introducing sustainable development topics into computer science education: design and evaluation of the Eco JSity game. Sustainability.

[21] Xiao Y, Jiang C, Fang X (2020). HCI in Games. HCII 2020. Lecture Notes in Computer Science.

[22] Cai S, Liu C, Wang T, Liu E, Liang JC (2021). Effects of learning physics using augmented reality on students’ self‐efficacy and conceptions of learning. Brit J Educ Technol.

[23] Lu SJ, Liu YC (2015). Integrating augmented reality technology to enhance children’s learning in marine education. Environ Educ Res.

[24] Wang Y, Ko E, Wang H (2022). Augmented reality (AR) app use in the beauty product industry and consumer purchase intention. Asia Pacific Journal of Marketing and Logistics.

[25] Lu SJ, Liu YC, Chen PJ, Hsieh MR (2020). Evaluation of AR embedded physical puzzle game on students’ learning achievement and motivation on elementary natural science. Interactive Learning Environments.

[26] van der Graaf J, Segers E, Verhoeven L (2016). Discovering the laws of physics with a serious game in kindergarten. Computers & Education.

[27] Jamonnak S, Cheng E (2017). Little Botany: a mobile game utilizing data integration to enhance plant science education. International Journal of Computer Games Technology.

[28] Chen MHM, Tsai ST, Chang CC (2019). Effects of game-based instruction on the results of primary school children taking a natural science course. Education Sciences.

[29] Liew CW, Treagust DF (1998). The effectiveness of predict-observe-explain tasks in diagnosing students’ understanding of science and in identifying their levels of achievement. ERIC.

[30] Bayrak BK (2013). Using two-tier test to identify primary students’ conceptual understanding and alternative conceptions in acid base. MIJE.

[31] Li FY, Hwang GJ, Chen PY, Lin YJ (2021). Effects of a concept mapping-based two-tier test strategy on students’ digital game-based learning performances and behavioral patterns. Computers & Education.

[32] Vos N, van der Meijden H, Denessen E (2011). Effects of constructing versus playing an educational game on student motivation and deep learning strategy use. Computers & Education.

[33] Ryan RM, Deci EL (2000). Self-determination theory and the facilitation of intrinsic motivation, social development, and well-being. Am Psychol.

[34] Kohlstedt SG (2010). Teaching Children Science: Hands-On Nature Study in North America, 1890-1930.

[35] Cheng KH, Tsai CC (2016). The interaction of child–parent shared reading with an augmented reality (AR) picture book and parents’ conceptions of AR learning. Br J Educ Technol.

[36] Pellas N, Fotaris P, Kazanidis I, Wells D (2019). Augmenting the learning experience in primary and secondary school education: a systematic review of recent trends in augmented reality game-based learning. Virtual Real.

[37] Thomas BH (2012). A survey of visual, mixed, and augmented reality gaming. Computers in Entertainment.

[38] Lan R, Sun X, Liu B, Zaphiris P, Ioannou A (2022). Learning and Collaboration Technologies. Novel Technological Environments. HCII 2022. Lecture Notes in Computer Science, vol 13329.

[39] López-Martínez A, Meroño L, Cánovas-López M, García-de-Alcaraz A, Martínez-Aranda LM (2022). Using gamified strategies in higher education: relationship between intrinsic motivation and contextual variables. Sustainability.

