# Supplementary material for: An Augmented Reality Serious Game for Children’s Optical Science Education: Randomized Controlled Trial
Source: JMIR Serious Games. 2024 Feb 1;12:e47807. doi: 10.2196/47807 (PMC10879978; doi:10.2196/47807)

Multimedia Appendix 2

Table S1. POE test questions (pre and post experiment)

| Pre- experiment | Question | Criteria for determination |
| --- | --- | --- |
| Animal Vision | Can birds see nectar that we can't? Why? | Knowing that birds can absorb specific kinds of light waves so they can see nectar |
|  | If a dog sees a green leather ball, can he retrieve a red leather ball without any problems? Why? | Knowing that dogs can't tell the difference between red and green |
| Light Transmission | Does light encountering glass happen to turn? Why does this happen? | Knew it would run somewhere else; predicted correct position of light reflection after changing position |
|  | Does light turn when it meets a lens? Why does it change in this way? | Knew it would run somewhere else; predicted correct position of light reflection after changing position |
|  | Does your shadow appear when you turn on the overhead fluorescent light? Where will the shadow appear? What shape is it? | Knowing that a shadow will appear  Predicts shadow position and shape correctly |
|  | Is there a difference between a shadow and a place without a shadow? Why is it different? | Understanding the causes and phenomena of shadows |
| Color-Light Mixing | Is green light a primary color light? What color is red and green together? | Know that light comes in different colors; know that color light composes different colors |
|  | Does green light promote plant growth? Why | Know the role of different colors of light for plant growth |

| Post- experiment | Question | Criteria for determination |
| --- | --- | --- |
| Animal Vision | Can an owl see other critters in the dark of night? Why? | Know that owls can absorb specific types of light waves, resulting in good night vision |
|  | If the dog sees the red ball, can he retrieve the red ball without any problems? Why? | Knowing that dogs can't tell the difference between red and green |
| Light Transmission | Does light turn when it meets ice? Why does it change like that? | Knows that light turns; after changing position, predicts the correct position of light reflection |
|  | Does light turn when it encounters a plane mirror? Why does it change in this way? | Knowing that a shadow will appear  Predicts shadow position and shape correctly |
|  | Will your shadow appear when you turn on the flashlight light on the table? Where will the shadow appear? What shape is it? | Knowing that a shadow will appear  Predicts shadow position and shape correctly |
|  | What color is the shadow? Why is it that color? | Understanding the causes and phenomena of shadows |
| Color-Light Mixing | Is yellow light a primary color light? What color is yellow and red together | Know that light comes in different colors; know that color light composes different colors |
|  | Does red light promote plant growth? Why | Know the role of different colors of light for plant growth |

Table S2. IMI scale

| COMP |
| --- |
| I think I was good in making/playing this game |
| I think I did pretty well in making this game, compared to others |
| I am satisfied with my performance while making the game |
| I was pretty skilled at making this game |
| I think I was pretty good in making this game |
| INT-ENJ |
| I think making this game was quite enjoyable |
| I think making this game was interesting |
| I think making this game was fun |
| While I was making the game, I often thought about how much I enjoyed it |
| I think making this game was boring |
| EFF-IMP |
| I did my best while I was making the game |
| I tried very hard to do well in making this game |
| It was important to me to do well in making this game |
| I put much effort in making this game |
| TEN-PRESS |
| I felt very tense while doing this activity |
| I did not feel nervous at all while doing this. |
| I was anxious while working on this task |
| I felt pressured while doing these. |

Interest - Enjoyment (INT-ENJ), Perceptual Competence (COMP), Effort - Importance (EFF-IMP) and Tension-Pressure (TEN-PRESS)

Table S3. Normality-test of two groups

|  |  | Pre | | | Aft | | |
| --- | --- | --- | --- | --- | --- | --- | --- |
|  |  | Mean ± Sd | z | p | Mean ± Sd | z | p |
| experimental group (n=18) | Pre- Animal Vision | 1.83±1.10 | 0.848 | 0.008 | 2.33±1.14 | 0.854 | 0.010 |
|  | Pre- Light Transmission | 2.83±1.72 | 0.878 | 0.025 | 4.44±1.76 | 0.937 | 0.262 |
|  | Pre- Color-Light Mixing | 1.50±1.09 | 0.878 | 0.024 | 2.39±1.24 | 0.910 | 0.086 |
|  | Pre- POE Score | 6.17±2.28 | 0.959 | 0.584 | 9.17±2.48 | 0.864 | 0.014 |
| Control group (n=18) | Pre- Animal Vision | 1.94±1.21 | 0.901 | 0.060 | 2.17±0.99 | 0.914 | 0.099 |
|  | Pre- Light Transmission | 2.67±2.20 | 0.926 | 0.165 | 3.00±1.88 | 0.806 | 0.002 |
|  | Pre- Color-Light Mixing | 1.94±1.16 | 0.897 | 0.051 | 2.50±1.04 | 0.896 | 0.049 |
|  | Pre- POE Score | 6.56±2.12 | 0.934 | 0.232 | 7.67±1.71 | 0.924 | 0.155 |

Table S4. Strengths and weaknesses in comparison with other studies

| Author | Content | Technology | User | Interaction | Advantage | Disadvantage |
| --- | --- | --- | --- | --- | --- | --- |
| Our design | Optics | AR | Children | Interaction with various optical phenomena in 3D space | Rendering of lights with 3D properties  Multiple interactive feedback | Not conducive to focus |
| Laine, et al [9] (2016) | Geometric | AR | Children | Explore the virtual environment with the main character | Increase intrinsic motivation  Creating pedagogically meaningful and versatile challenges | Only the conceptual feasibility of SSAR was investigated, lacking validation |
| Tarng et al [14] (2018) | Astrology | AR | adolescents | The mobile device displays the virtual sun in the same direction as the real sun | Combination of sensor functions to simulate the sun's path | Lack of data support to prove effectiveness |
| Nkadimeng and Ankiewicz [19] (2022) | Chemotherapy | Mobile 2D games | adolescents | Support for collaboration (visiting each other's atomic structure worlds) | Collaborative learning is possible to increase participation | Not conducive to focus |
| Swacha et al [20] (2021) | Nature and the environment system | Mobile 2D games | university student | Role-playing to solve problems through programming tasks that compute environmental loads. | Interesting and purposeful combination of lessons | Less playful  Unnecessary distraction of students from course topics  Lack of measurement of educational outcomes |
| Xiao and Jiang [21] (2020) | Acoustical | Mobile 2D games | Children | Explore knowledge of sound propagation | Graphic design to visualize abstract sounds | Single form of interaction |
| Cai et al [22] (2021) | Photoelectric effect | AR | adolescents | Simulation of real-life photoelectric effect experiments | Highly accurate reproduction of optoelectronic model features and interactions in real environments | Low interest  Primarily examines self-efficacy for learning, with no elaboration of learning outcomes |
| Lu and Liu [23] (2015) | Water ecology and marine environment | AR | Children | Popular science narrative storytelling, somatosensory interaction | A complete science process of play-learning-testing. Targeted learning in a teaching environment through projector equipment, combined with competitions to increase fun | Restricted environment, need to play in a classroom, and need guidance from professionals |
| Wang et al [24] (2022) | Magnetic | AR | adolescents | Manipulating magnets in AR environments to visualize magnetic induction lines | Incorporate contest quizzes to increase student-teacher and student-student interaction rates | Lack of gameplay and few teaching scenarios |
| Lu et al [25] (2020) | Dinosaur period ecology, dinosaur fossils | AR | Children | Identify dinosaur picture card material and generate puzzles to answer based on the picture cards | Utilizing the framework of the taxonomy, the learning content and games utilize "memorize-understand-apply" to deepen children's grasp of knowledge. | No significant effect on motivation to learn |
| van der Graaf et al [26] (2016) | Physical law | iPad 2D game | Children | Manipulating facilities in the environment, such as placing weights on one end of a seesaw, to accomplish tasks | Children independently explore in play scenarios, generating non-verbal reasoning and enhancing the efficiency of knowledge acquisition through play | No analysis of learning outcomes |
| Jamonnak and Cheng [27] (2017) | Botanic | ipad 2D game | Children | Growing vegetable plants in the game, observing the effects of weather changes on plant growth, and maintaining plant health on a daily basis | Simulate weather changes based on the weather at the user's location | Only usability testing was done, no analysis of learning outcomes |
| Chen et al [28] (2019) | Carbon emission | Mobile /computer games | Children | Characters interact in game scenarios, calculate daily carbon emissions, learn and complete interactive exercises | Stage-by-stage control of children's learning, with tasks designed to unlock the next stage of play. | Limited game-based diversity of practice questions reduces children's motivation to learn |

Figure S1. Content of the AR game


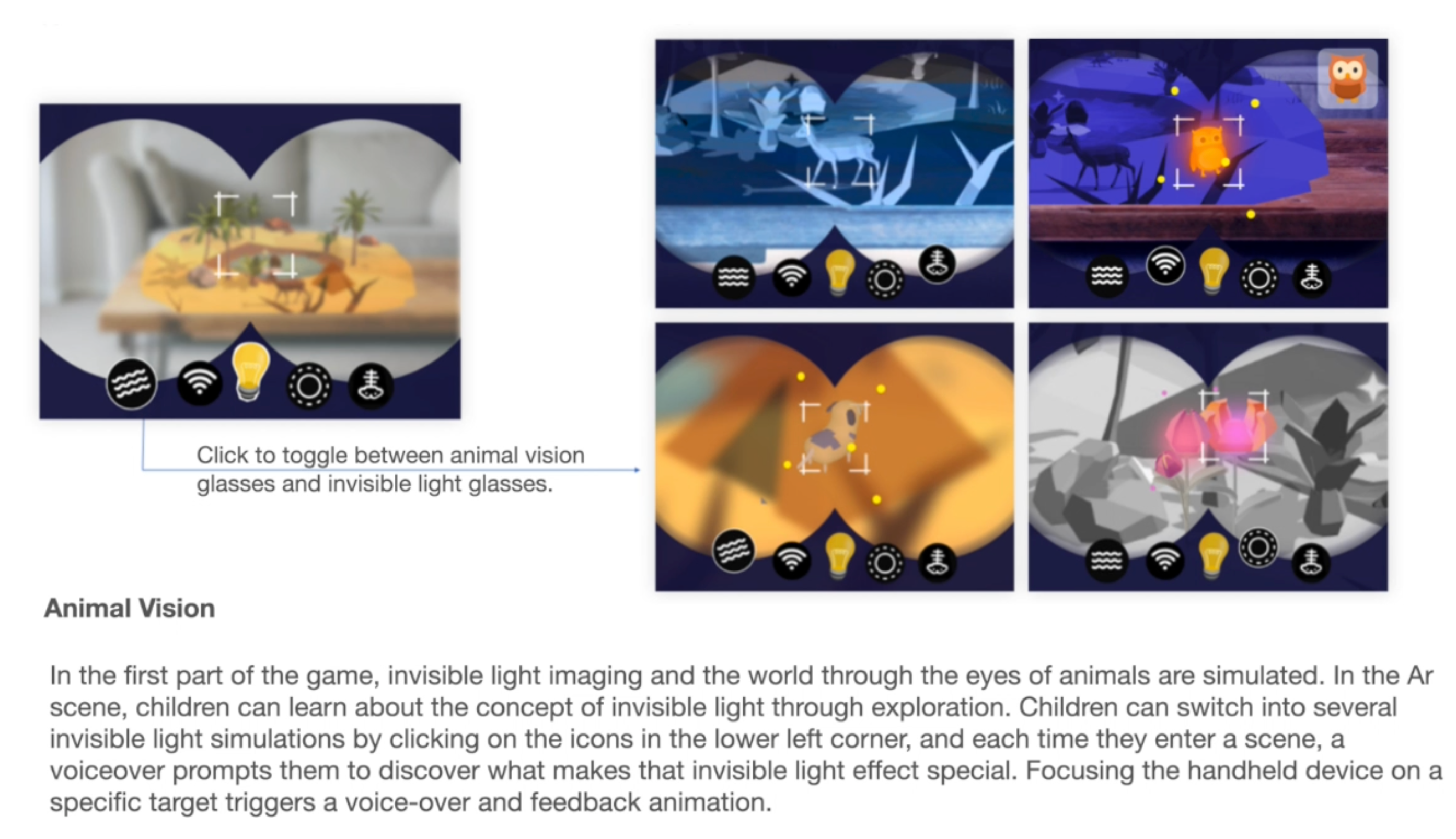


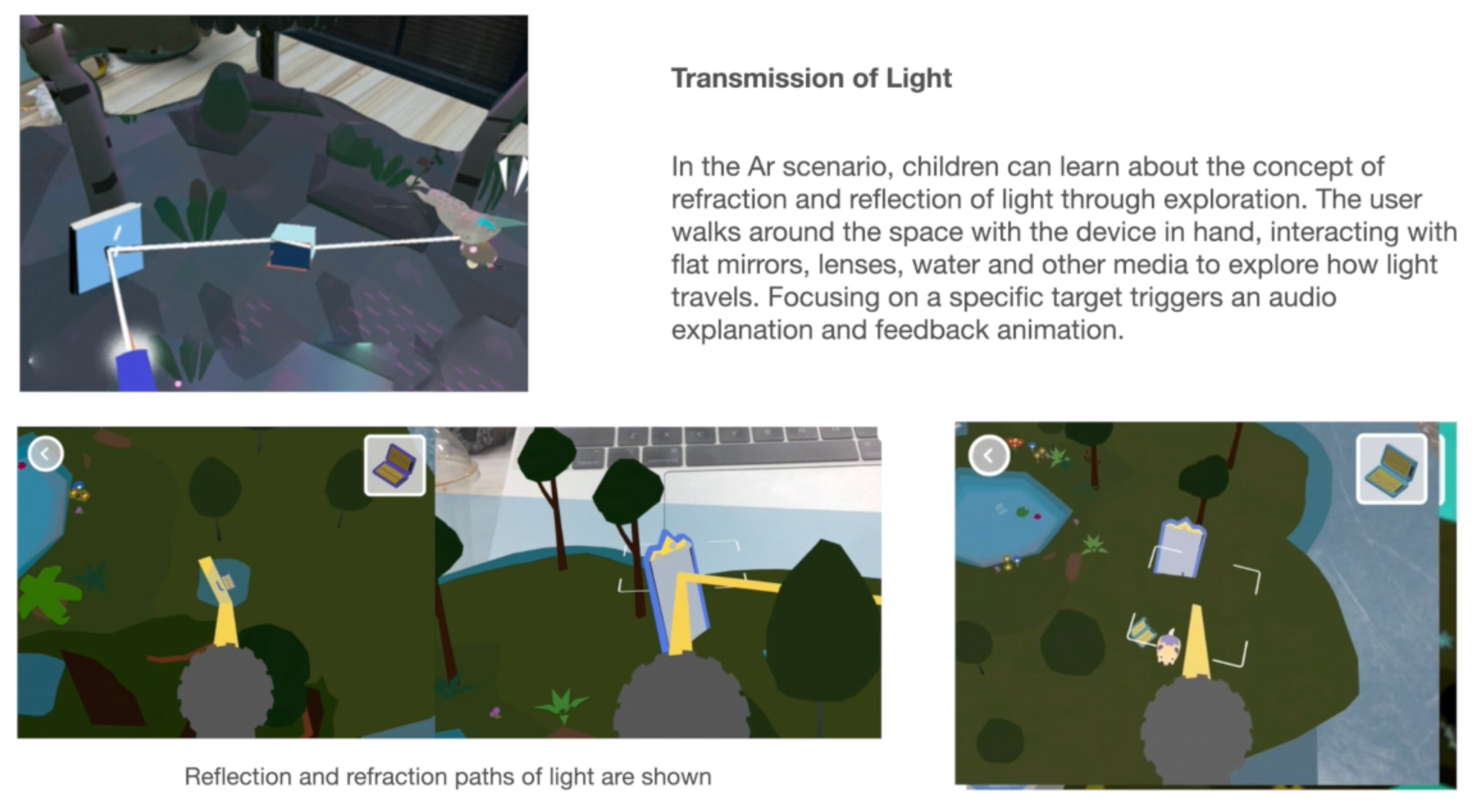


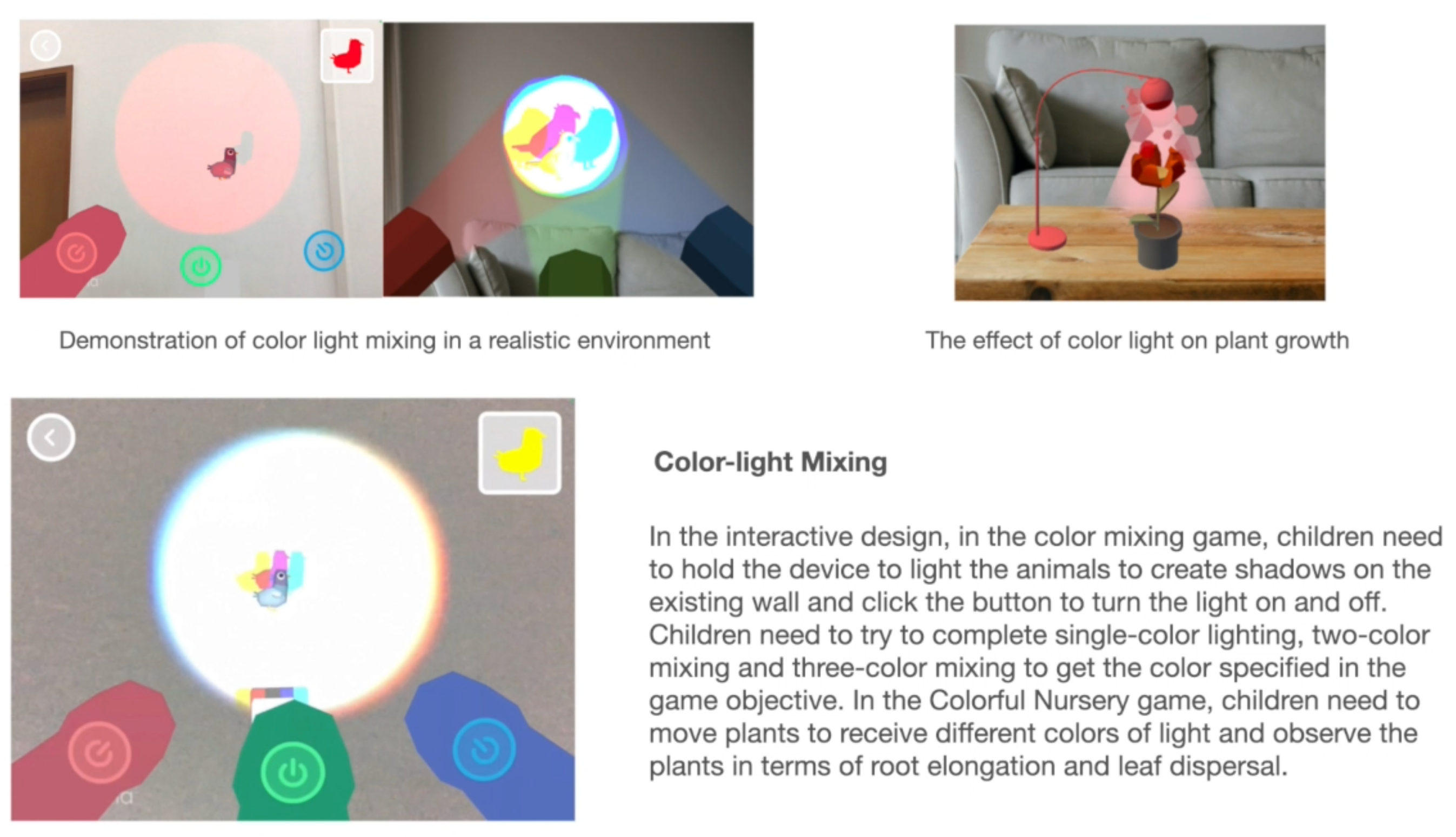


Figure S2. Difference between two games


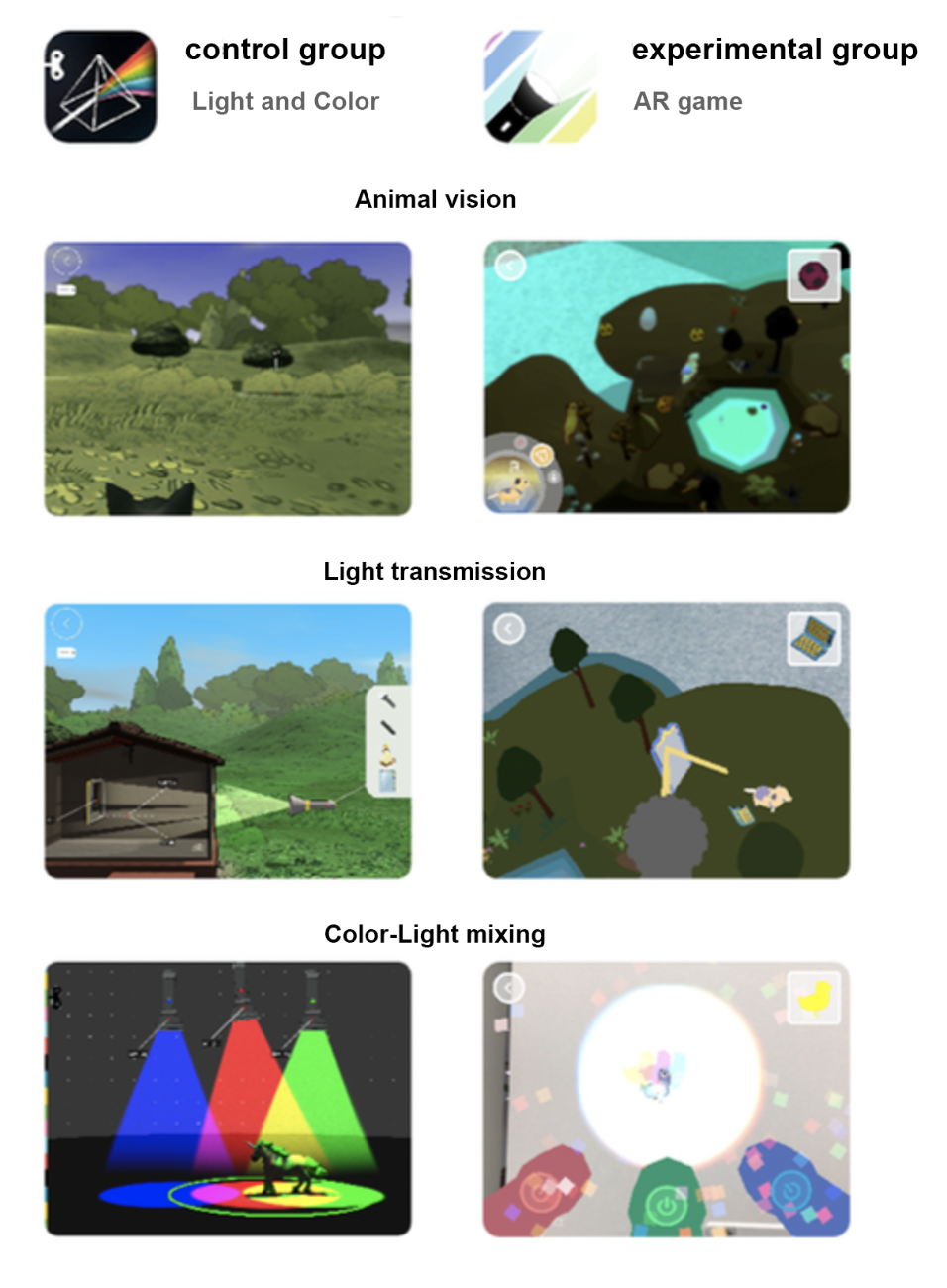

Supplement: Multimedia Appendix 2 [file games-v12-e47807-s002.docx]
